# Mechanistic Understanding of Toxicity from Nanocatalysts

**DOI:** 10.3390/ijms150813967

**Published:** 2014-08-12

**Authors:** Cuijuan Jiang, Jianbo Jia, Shumei Zhai

**Affiliations:** School of Chemistry and Chemical Engineering, Shandong University, Jinan 250100, China; E-Mails: cjjiang@sdu.edu.cn (C.J.); jiajianbo03@gmail.com (J.J.)

**Keywords:** nanocatalysts, environmental remediation, safety concerns, toxicology, mechanism

## Abstract

Nanoparticle-based catalysts, or nanocatalysts, have been applied in various industrial sectors, including refineries, petrochemical plants, the pharmaceutical industry, the chemical industry, food processing, and environmental remediation. As a result, there is an increasing risk of human exposure to nanocatalysts. This review evaluates the toxicity of popular nanocatalysts applied in industrial processes in cell and animal models. The molecular mechanisms associated with such nanotoxicity are emphasized to reveal common toxicity-inducing pathways from various nanocatalysts and the uniqueness of each specific nanocatalyst.

## 1. Introduction

Homogeneous catalysis and heterogeneous catalysis each have their own advantages and disadvantages. For example, homogeneous catalysis exhibits a high reactivity, a good selectivity, and an excellent reaction yield; however, it is easier to manage heterogeneous catalytic reactions and to remove the catalysts from the reaction mixtures after these reactions. Nonetheless, there is considerable difficulty in product/catalyst separation in homogeneous catalysis and decreased overall catalytic efficiency in heterogeneous catalysis [1,2]. Therefore, new catalyst systems are highly desirable.

**Figure 1:**
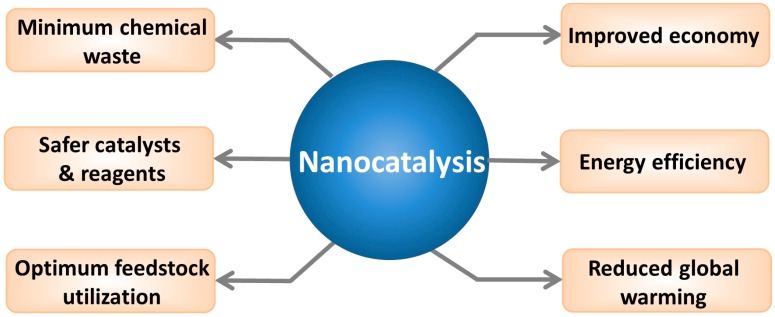
The expected benefits of nanocatalysis. Reprinted from [2] with permission from Wiley-VCH Verlag GmbH & Co. KGaA, Copyright 2013.

Because of their high surface area, nanomaterials have emerged to bridge the gap between homogeneous and heterogeneous catalysis approaches. Common nanocatalysts include carbon-based nanomaterials (fullerenes [3], grapheme [4] and carbon nanotubes [5]), metals (iron [6], silver [7], gold [8], and cobalt [9]), oxides (zinc oxide [10], titanium dioxide [11], and silicon dioxide [12]), and other nanomaterials (such as quantum dots [13]). Compared to regular catalysts, nanocatalysts possess certain unique advantages, such as an enhanced mixing with reactants and easy separation from the reaction mixture due to their insolubility in various solvents. Moreover, it is easier to regulate the catalytic activity and selectivity of nanocatalysts by tailoring the chemical and physical properties of the catalysts [1]. Indeed, the use of nanocatalysts may lead to improved energy efficiency and economy. For instance, a hydrotalcite nanocatalyst was found to be stable, inexpensive, highly active, and selective for the hydrolysis of cellulose to glucose [14]. Much less chemical waste and optimized feedstock utilization have also been achieved using nanocatalysts [2]. In view of the numerous potential benefits (Figure 1), nanocatalysts have been applied in various areas, including refineries [15], petrochemical plants [15], the pharmaceutical industry [16], the chemical industry [17], food processing [18], and environmental applications [7]. According to Global Industry Analysts, the global nanocatalyst market is projected to reach 6 billion US dollars by 2015 [19].

When the size of a material is reduced to the nanoscale, the material often exhibits unusual physicochemical properties. In addition to improved catalytic activity, conductivity, reactivity, and optical sensitivity, nanosized materials also exhibit increased uptake in and interactions with biological tissues and can affect biological functions. Indeed, recent studies on potential nanotoxicity to human health and the eco-environment have drawn attention from both government agencies and the general public [20,21]. With the increasing commercialization of, and mounting human exposure to nanocatalysts, a safety assessment of these materials is needed. To facilitate such efforts, we herein survey the known toxic effects of several nanocatalysts, mainly used in environmental remediation.

## 2. Carbon-Based Nanomaterials

Three well-known carbon-based nanomaterials (CBNs) are fullerenes [22], carbon nanotubes (CNTs), including single-walled and multi-walled CNTs (SWCNTs and MWCNTs) [23], and grapheme [24]. Due to their excellent electrochemical stability, conductivity, high surface area, and mechanical strength, C60 nanoparticles and CNTs were found to enhance catalytic activity when used as catalyst carriers in nanocatalysis [23,25]. CNTs have been shown to be good support materials in various heterocatalytic reactions, such as in NH_3_ synthesis [26], cinnamaldehyde hydrogenation [27], and methanol oxidation [28]. In environmental remediation, Ru/CNT nanocomposites have exhibited a high activity and stability in catalyzing ammonia decomposition for the generation of CO_x_-free hydrogen (x = 1 and 2) [5]. Fe-embedded graphenes were shown to have a good catalytic activity for CO oxidation by computation, suggesting the potential application of metal-graphene systems in solving the growing environmental problems caused by CO [3]. This potential has been confirmed in an experiment showing that a graphene-supported palladium catalyst exhibited superior activity and stability with regard to CO oxidation [29].

Controversial results have been obtained regarding the cytotoxicity of C60 nanoparticles in various cell lines [30]. No significant cytotoxic responses to pristine C60 nanoparticles were observed in murine macrophages [31], human monocyte-derived macrophages [31,32], and Guinea pig alveolar macrophages [33]. In contrast, C60 nanoparticles were found to be cytotoxic in other cell lines, such as human dermal fibroblasts (HDFs) [34,35], human liver carcinoma cells (HepG2) [34,35], human epidermal keratinocytes (HEK) [36], mouse L929 fibrosarcoma cells [37], rat C6 glioma cells [37], and human U251 glioma cells [37]. Although the surface functionalization of C60 particles may partially alleviate their cytotoxicity and reduce reactive oxygen species (ROS) generation [34], polyhydroxylated fullerenes can still induce photooxidative stress in human cells [38,39].

In contrast to C60 nanoparticles, both SWCNTs and MWCNTs consistently show cytotoxic effects at high concentrations. Cytotoxicity induced by MWCNTs or SWCNTs has been observed in HEK [40], human embryo kidney cells (HEK293) [41], rat alveolar macrophage cells (NR8383) [42], human alveolar epithelial cells (A549) [42], human skin fibroblasts (HSF42) [43], and human embryonic lung fibroblasts (IMR-90) [43]. Comparative studies have also shown that SWCNTs are more toxic than MWCNTs and C60 nanoparticles [33,42,44]. Although pristine SWCNTs are cytotoxic, these toxic effects can be lessened by surface functionalization, with the material becoming more soluble and less cytotoxic as the degree of sidewall functionalization increases [45].

To date, several studies have been carried out investigating the *in vitro* cytotoxicity of graphene and its derivatives. Generally, graphene oxide (GO) were shown to cause a dose- and time-dependent cytotoxicity in various cells including A549, Henrietta Lacks cells (HeLa), National Institute of Health 3T3 mouse fibroblast cells (NIH-3T3), Sloan Kettering breast cancer cells (SKBR3), Michigan cancer foundation-7 breast cancer cells (MCF7), human neuroblastoma SH-SY5Y cells and even normal human lung cells (BEAS-2B) [46,47,48,49]. Size and surface coating are also factors that influence the cytotoxicity of graphene. Reduced graphene oxide nanoplatelets (rGONPs) with average lateral dimensions (ALDs) of 11 ± 4 nm caused significant cell destruction at the concentration of 1.0 μg/mL, while the rGONPs with ALDs of 3.8 ± 0.4 μm exhibited significant cytotoxic effects only at high concentrations of 100 μg/mL [50]. Furthermore, surfaces coated with chitosan significantly reduce the haemolytic activity of GO to human erythrocytes (RBCs) [51].

*In vivo* toxicity evaluations of C60 nanoparticles have primarily been performed in rat and fish. When intratracheal instillation and inhalation were used, neither pristine nor functionalized fullerenes caused significant histopathologic abnormalities, with the exception of a slight and transient lung inflammation [52,53,54,55]. In contrast, using intraperitoneal or intravenous administration, both pristine and derivatized C60 nanoparticles showed toxic effects at high doses (>500 mg/kg) and antioxidant tissue-protective effects at lower doses [56]. However, no significant toxicity was observed when animals were exposed to C60 nanoparticles through the oral, dermal, or ocular route [57,58,59].

**Figure 2:**
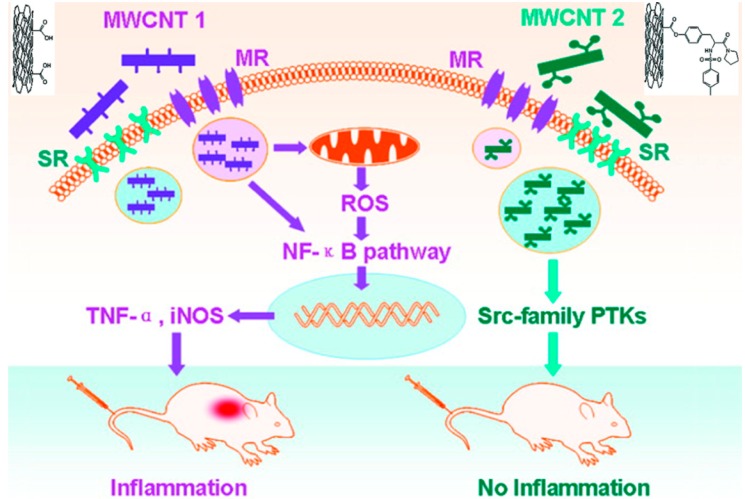
Surface chemistry modification of MWCNT (multi-walled carbon nanotube) 2 significantly alleviated NF-κB activation and reduced the immunotoxicity caused by unmodified MWCNT 1. Adapted from [71] with permission from the American Chemical Society, Copyright 2011.

MWCNTs and SWCNTs induced pulmonary inflammatory responses and granuloma formation after intratracheal instillation in animal models [60,61,62], though the results occasionally varied, depending on the animal model used. Dose-dependent granuloma formation was induced by SWCNTs in mice [61,63], and a series of multifocal granulomas was observed in rats [64]. Inhalation exposure was also used in such investigations. Wistar rats showed no systemic toxicity beyond pronounced multifocal granulomatous inflammation after inhalation exposure to MWCNTs at 0.5 or 2.5 mg/m^3^ [65]. In contrast, aside from certain immune system alterations, no significant lung inflammation or tissue damage was observed in adult male C57BL/6 mice after inhalation of MWCNTs at 0.3, 1, or 5 mg/m^3^ [66]. This disparity may be attributed to the use of different preparations of MWCNTs and different animal models. In addition to respiratory toxicity, CNTs were repeatedly shown to cause perturbations of the immune system [67,68,69,70], and such immunotoxicity can be reduced by surface chemistry modification of MWCNTs, as demonstrated by our laboratory [71]. A modification of the chemical structure of the surface ligands of MWCNTs (14 mg/kg via intravenously administration) was shown to increase the binding of nanotubes to scavenger receptors and reduce NF-κB activation and associated inflammation in mice (Figure 2). Furthermore, repeated administration of MWCNTs (5 doses over 13 days at 5 mg/kg per dose) to male BALB/c mice caused reversible testis damage and oxidative stress in the testes without affecting fertility, suggesting a potential reproductive toxicity for MWCNTs [72].

*In vivo* toxicity assessment of graphene and its derivatives has also been investigated in animal models. After intravenous administration, GO was found mainly deposited in the lungs, and retained for a long time [73]. GO under low dose (<0.25 mg per mice) did not exhibit obvious toxicity to Kunming mice; however, at high concentration (0.4 mg per mice), GO exhibited chronic toxicity, which including mice death and lung granuloma formation [74]. Distribution and biocompatibility of GO were considered to be regulated via surface functionalization. In detail, GO exhibited low uptake in the reticuloendothelial system (RES) [73]; while dextran functionalized graphene was found accumulated in the RES including liver and spleen after intravenous injection [75]. On the other hand, PEGylated GO exhibited low uptake by the RES, highly efficient tumor passive targeting, and no obvious side effect on the injected mice [76].

Among the various hypothesized nanotoxicity mechanisms, ROS generation is a generally accepted mechanism [77,78]. SWCNTs also mediate an upregulation of apoptosis-associated genes and a downregulation of cell cycle-associated genes [41]. These events may explain the observations that CNTs often induce cell cycle arrest and cell apoptosis. The exposure of BEAS-2B cells to MWCNTs was shown to result in NF-κB signaling pathway activation, enhanced phosphorylation of mitogen-activated protein kinase (MAPK) pathway components, and increased production of proinflammatory cytokines [79]. SWCNTs were found to induce toxicity in BEAS-2B cells through perturbations of the AP-1, NF-κB, and MAPK pathways and the activation of caspase-3, caspase-7, and poly (ADP-ribose) polymerase-1 (PARP-1) [77]. In our previous study, MWCNTs were demonstrated to suppress the bone morphogenetic protein (BMP) signaling pathway, which led to the downregulation of Id protein expression and upregulation of p21 expression, leading to cell cycle arrest [51]. We also demonstrated that, by binding to the BMP receptor 2, MWCNTs regulated basic helix-loop-helix (bHLH) transcription factors via BMP signaling suppression, leading to enhanced cell differentiation and inhibited apoptosis in mouse myoblast cells [73]. A more important finding was that surface-modified MWCNTs [80] regulated cell differentiation to various controllable levels by regulating BMP signaling [73].

In conclusion, all these three kinds of CBNs can be toxic *in vitro* and *in vivo* at certain concentrations, and surface functionalization appears to play an important role in reducing such toxic effects. Toxicity due to these CBNs has been correlated with ROS generation and the activation of molecular signaling pathways and regulation of expression of key proteins.

## 3. Titanium Dioxide Nanoparticles

Due to their photocatalytic activity, titanium dioxide nanoparticles (TNPs) have been widely applied in environmental remediation [81,82,83,84]. Under light, both micro- and nano-structured TiO_2_ can promote the breakdown of environmental pollutants at room temperature. As a result, TiO_2_ micro- and nano-particles have been employed in various processes, such as water treatment, gas treatment, organic pollutant degradation, and pollutant removal [11,85,86]. In food processing, nanostructured TiO_2_ photocatalysts combined with Ultraviolet A (UVA) irradiation have been used to eliminate pathogenic microorganisms in food-contacting surfaces [18]. Although TNPs have long been considered nontoxic [87], with the increasing commercialization of TNP-related products, concerns about the possible toxicity of TNP exposure have motivated further research efforts.

Cytotoxicity assessments of TNPs have been performed in various cells, and the cell type does appear to play a role in TNP-induced cytotoxicity. TNPs have shown very little cytotoxicity in primary human peripheral blood mononuclear cells (PBMCs) [88], monocyte-derived dendritic cells (MDDCs) [88], and rat peripheral blood neutrophils [89] at concentrations up to 100 μg/mL. A similar result was obtained in primary cultures of human hematopoietic progenitor cells [90]. However, a dose-dependent cytotoxicity was observed for TNPs in other cell lines, such as human skin fibroblasts [91], mouse fibroblasts (L929) [92], rabbit erythrocytes [93], and human B-cell lymphoblastoid cells [94]. Moreover, genotoxicity studies in various cell lines have shown that TNPs cause DNA damage and increase the mutation frequency [94,95,96], and more serious DNA damage has been detected under ultraviolet radiation [97]. Therefore, the cell type appears to be a factor influencing the cytotoxicity of TNPs, a notion that was also confirmed in a study of the responses of several eukaryotic cells to TNP exposure [95].

The *in vivo* toxicity of TNPs has been investigated in various aquatic organisms and in animal models. A general growth inhibition was observed in freshwater algae, and variable EC_50_ values, ranging from 5.83 [98] to 241 mg/L [48] TNPs, were reported in *Pseudokirchneriella subcapitata*. The toxicological effects of TNPs on the freshwater invertebrate *Daphnia magna* were similar to the effects on freshwater algae [99], and a high LC_50_ value (500 mg/L and higher) was found in fish [100]. In addition to ecotoxicological assessments in aquatic organisms, the biodistribution and toxicity of TNPs in rats and mice were also evaluated. After a single intravenous injection (5 mg/kg body weight) in Wistar rats, TNPs predominantly accumulated in the liver, in addition to the spleen, lung, and kidney [101]. No obvious toxic effect, immune response, or change in organ function was observed. The order of TNP accumulation in CD-1 (Imprinting Control Region, ICR) mice after an abdominal cavity injection was liver > kidneys > spleen > lung > brain > heart [102]. At a very high dose (150 mg/kg body weight) via intraperitoneal injection, TNPs caused serious damage to the liver, kidney, and myocardium and resulted in liver DNA cleavage in mice [102,103]. An intratracheal instillation (0.5, 5, or 50 mg/kg) of TNPs of various sizes (5, 21, and 50 nm) into Sprague-Dawley rats caused dose-dependent inflammatory lesions, as observed by histopathologic examination [104], work that also showed that the particle size and exposure dose played important roles in the pulmonary toxicity of TNPs. To assess the toxicity of TNPs in sensitive populations, mice with ovalbumin (OVA)-induced airway inflammation were investigated, with TNP exposure via a single or repeated inhalations resulting in respiratory diseases and dose- and time-dependent toxicity [105].

**Figure 3:**
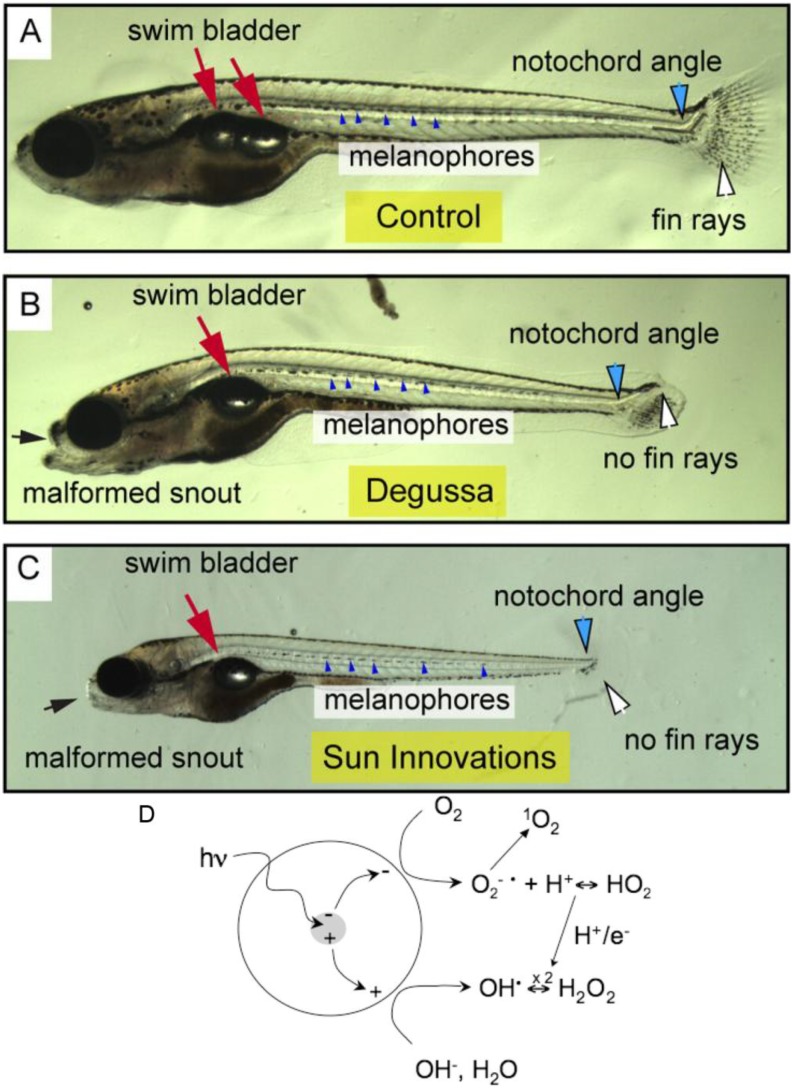
TNP (titanium dioxide nanoparticle) exposure-induced photodependent delays in development, decreased growth, and tissue malformation and possible mechanisms. Fish were exposed to illumination in all cases with (**A**) water as a control; (**B**) Degussa TNPs (1 ng/mL); or (**C**) Sun Innovations TNPs (1 ng/mL). Representative effects of the TNPs were indicated. The red arrows show the normal bi-lobed swim bladder in the control and the single-lobed swim bladder in the treated fish. The small blue points indicate the row of developing melanophores making an unbroken line in the normal fish, and underdeveloped in the treated fish. Fin rays in the normal fish are indicated by a white arrow, and the angle of the notochord as it intersects the caudal fin is indicated by a black line and blue arrow. The black arrow indicates the snout shortening and related craniofacial malformations caused by treatment. Possible mechanisms of illumination-induced ROS (reactive oxygen species) generation were performed in (**D**). Adapted from [110,111] with permissions from the American Chemical Society, Copyright 2009, 2013.

Toxicity caused by TNPs has been attributed to ROS generation*,* and *in vitro* studies have shown that TNPs caused oxidative stress in various human cell lines, such as human skin fibroblast cells [106], human bronchial epithelial cells (BEAS-2B) [107], and MG63 osteoblast-like cells [108]. Dose-dependent ROS generation and lactate dehydrogenase (LDH) production were observed in mouse fibroblast cells (L929), suggesting the involvement of ROS in the cytotoxicity of TNPs [92]. Similar results have been obtained in fish and algae [98,109]. Furthermore, light can play an important role in toxicity due to the photocatalytic property of TNPs. A separation of charge was induced in TNPs under sunlight or artificial light (Figure 3D), whereby the valence-band holes generated at the surface of the excited particle abstracted electrons from water and/or hydroxyl ions, leading to the generation of hydroxyl radicals (OH•) [110]. Thus, TNPs exhibited enhanced antibacterial properties against *Escherichia coli* K12 under light. At the same time, TNPs became more toxic to zebrafish embryos [111,112]. In buffered water consisting of reverse osmosis purified water and 60 mg/L Instant Ocean Salts, the exposure of zebrafish embryos to illumination and 1 ng/mL TNPs led to a failure to progress through metamorphosis and a reduced overall size and certain defects in the adult fish (Figure 3A–C) [111]. However, in a real conditions in river or lake, high concentrations of organic contaminants exit and these organics are preferentially decomposed by photocatalysis. The addition of dissolved organic matter (DOM) in “fish water” increased TNPs suspension stability and reduced levels of Ti associated with fish [113]. However, photodegradation products of DOM produced in the presence of TNPs caused higher levels of oxidative DNA damage, resulting in higher mortality of zebrafish embryos on the contrary.

In brief, the toxicity of TNPs has been investigated in a variety of models under various experimental conditions. Toxicity was observed in aquatic organisms and in animals and was further enhanced under illumination. However, the results were variable between various models, which may be attributed to variation in the materials used and the experimental conditions, suggesting the necessity of formulating more comprehensive and quantitative protocols for evaluating nanotoxicity in general.

## 4. Gold Nanoparticles

Gold colloids, or gold nanoparticles (GNPs), have unique properties, such as easy surface modification, chemical stability, and size- and shape-dependent optical and electronic features. GNPs have been widely used in chemistry, biology, and medicine [114] and have recently been used as catalysts for various oxidation reactions, such as aldehyde oxidation [115], alkene epoxidation [116], and the aerobic oxidation of alcohols [117]. In environmental remediation, nanocomposites, such as TiO_2_/Au and ZnO/Au nanostructures, have been explored for pollutant degradation [8,118]. Nanocatalysts involving GNPs also showed high efficiency in the oxidation of CO [119,120,121]. Because of the wide applications of GNPs in many fields, the toxicity of GNPs has accordingly been investigated.

**Figure 4:**
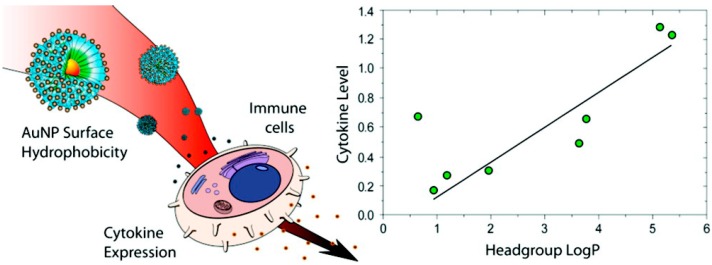
The surface hydrophobicity of GNPs (gold nanoparticles) dictates immune responses in splenocytes. Reprinted from [130] with permission from the American Chemical Society, Copyright 2012.

Several factors play important roles in determining the cytotoxicity of GNPs. Notably, surface properties have been shown to affect the biological interactions of GNPs [122]. In one study, polyethylenimine-modified GNPs exhibited excellent transfection efficiency in monkey kidney cells (COS-7) but also caused decreased cell viability [123]. However, GNPs with different surface charges have exhibited different cell uptake [124] and toxic effects [125,126] in cells. For example, positively charged GNPs were reported to modulate the cell membrane potential and cause cell membrane perturbations during cellular uptake. However, when their surfaces were functionalized with a peptide, GNPs entered cells and targeted the nucleus without decreasing cell viability [127]. Apart from the surface properties, the particle size is another important physical parameter that determines the cytotoxicity of GNPs. When triphenylphosphine derivative-stabilized GNPs ranging from 0.8 to 15 nm in diameter were added to HeLa cells, SK-Mel-28 melanoma cells, L929 mouse fibroblasts, and mouse monocytic/macrophage cells, the GNPs exerting the most cytotoxic effects had a diameter of 1.4 nm [128]. GNP-induced cytotoxicity was also found to depend on the cell type [129]. In addition to cytotoxicity, GNPs cause an immunological response in cells. For instance, the surface hydrophobicity of GNPs dictates the immune response of splenocytes [130], and there is a correlation between the hydrophobicity of GNP surface ligands and immune system activation (Figure 4).

Polyethylene glycol (PEG)-modified GNPs caused acute inflammation and apoptosis in the liver of mice after intravenous administration [131]. Moreover, PEG-coated GNPs of either 4 or 100 nm significantly altered gene expression in the mouse liver at 30 min after a single intravenous injection [132]. The GNP dose is important for the toxicity of the nanomaterial *in vivo*. No acute or subacute physiological damage was observed after the intraperitoneal administration of 13-nm GNPs at a dose up to 0.4 mg/kg/day for 8 days [133], whereas GNPs caused severe sickness in mice after daily intraperitoneal administration at 1.15 mg/kg/day for 14 days [134]. The particle size is also an important factor *in vivo*. GNPs of 3, 5, 50, or 100 nm at a dose of 8 mg/kg/week did not show harmful effects in mice. However, GNPs with a size ranging from 8 to 37 nm at the same dose caused an increase in Kupffer cells in the liver, a loss of structural integrity in the lungs, and the diffusion of white pulp in the spleen [134]. *In vivo* toxicity assessments of GNPs have also been performed in zebrafish embryos and were found to be more biocompatible with the embryos than silver nanoparticles [135,136].

The generation of ROS and reactive nitrogen species (RNS) is considered to be responsible for GNP-induced toxicity. GNPs were found to induce oxidative stress-related autophagy or necrosis in HeLa cells and MRC-5 human fetal lung fibroblasts [137,138]. The release of nitrogen oxide (NO) from endogenous *S*-nitroso adducts with thiol groups in blood serum was induced by GNPs [139]. GNPs also inhibited vascular endothelial growth factor (VEGF)-induced cell proliferation by affecting related signaling pathways [140,141]. Furthermore, both citrate-coated and antigen-conjugated GNPs stimulated the respiratory activity of macrophages and the activity of macrophage mitochondrial enzymes [142].

In summary, several factors, such as surface functionalization, particle size, cell type, and administration method, have been shown to affect the toxic effects of GNPs. GNP-induced toxicity may be related to ROS and RNS generation and certain signaling pathway perturbations; however, the detailed mechanism of nanotoxicity due to GNPs remains to be elucidated.

## 5. Silver Nanoparticles

In addition to their excellent antibacterial property, silver nanoparticles (Ag NPs) are good catalysts [143]. Similar to GNPs, Ag NPs catalyze a range of oxidation reactions, including epoxidation reactions, the selective oxidation of hydrocarbons, and the oxidation of alcohols and aldehydes [144]. Furthermore, Ag NPs strongly absorb visible-light and UV light due to the surface plasmon resonance (SPR) effect and the interband transition (the 4d to the 5sp) [7,100,145]. Therefore, Ag NPs exhibit enhanced photocatalytic activity on the surface of various supports, showing a potential application in pollutant degradation in environmental remediation [7,146,147]. Due to their wide applications, the potential risks of Ag NPs have caused widespread concern.

The exposure of keratinocytes and fibroblasts to extracts of an Ag NP-containing wound dressing was found to cause reduced mitochondrial metabolism and cell viability [148,149]. Similar results were obtained in human fibrosarcoma and skin carcinoma cells [150], and the exposure of human PBMCs to Ag NPs caused dose-dependent cytokine production [151]. Reduced mitochondrial function and an increased level of ROS were observed in mouse spermatogonial stem cells (C18-4) [152] and BRL 3A rat liver cells [153] after Ag NP exposure. Ag NPs with different surface functionalizations exhibit different toxic effects in various cell lines. For instance, polysaccharide surface-functionalized Ag NPs caused more severe DNA damage than non-functionalized Ag NPs in mouse embryonic stem (mES) cells [154]. Unmodified Ag NPs caused a dose-dependent decrease in cell viability in HEK, whereas no significant toxic effects were induced by functionalized Ag NPs [155]. Furthermore, a size-dependent cytotoxicity of Ag NPs was observed in several cell lines, including a mouse preosteoblast cell line (MC3T3-E1) [156], a rat adrenal medulla-derived cell line (PC12) [156], a human cervical cancer cell line (HeLa) [156], human liver carcinoma (HepG2) [50], human alveolar epithelial cells (A549) [50], human gastric cancer cells (SGC-7901) [50], human breast cancer cells (MCF-7) [50], and Chinese hamster ovary (CHO) cells, derived from the ovaries of Chinese hamsters [156].

Ag NPs were found to be distributed in the kidneys, liver, spleen, lung, and even brain in rats after inhalation [157,158], oral administration [159], or subcutaneous injection [160]. However, no significant toxic effects, except for a slight increase in neutral mucins, was observed after a 28-day inhalation exposure to Ag NPs (up to 1.32 × 10^6^ particles/cm^3^, 6 h/day, five times per week for 28 days) in Sprague-Dawley rats [161,162]. Nonetheless, Ag NPs compromised lung function and induced inflammation in the same model after prolonged inhalation exposure (up to 2.9 × 10^6^ particles/cm^3^, 6 h/day, for 90 days) [158]. In mice, Ag NP exposure via both inhalation (1.91 × 10^7^ particles/cm^3^, 6 h/day, 5 days/week for 2 weeks) and intraperitoneal injection (up to 1000 mg/kg) altered gene expression in the mouse brain [163,164]. Oral exposure to Ag NPs has also been investigated to simulate the ingestion of nanoparticles in food-related products. Acute oral exposure to Ag NPs (2.5 g/mouse) caused lymphocytic infiltration into the mouse liver and the alteration of the expression of genes related to apoptosis and inflammation [165]. In a 28-day oral-exposure study in Sprague-Dawley rats, Ag NPs caused dose-dependent alterations in the concentration of alkaline phosphatase, indicating liver damage [166]. Such changes were also observed in a 90-day oral exposure study in Fisher 344 rats [167]. In addition to mammalian animal models, several non-mammalian animal models, such as zebrafish, fruit flies, and rainbow trout, have been used to assess the toxic effects of Ag NPs. For instance, Ag NPs induced dose-dependent mortality and developmental abnormality in zebrafish embryos [168,169].

**Figure 5:**
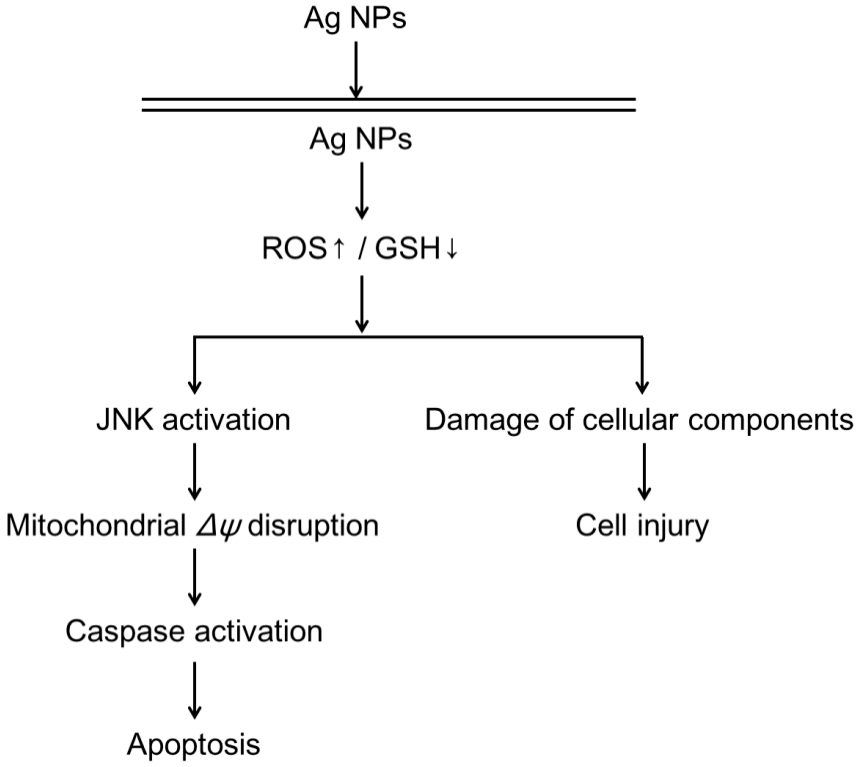
A proposed pathway for Ag NP-induced ROS generation, intracellular glutathione (GSH) depletion, damage to cellular components, and apoptosis. Reprinted from [174] with permission from Elsevier, Copyright 2011.

Studies have shown that cytotoxicity, DNA damage, and apoptosis induced by Ag NPs in various cell lines is likely to be mediated via the generation of oxidative stress [153,170,171]. Moreover, mitochondrial damage is another possible mechanism of Ag NP-induced toxicity. In several recent studies, Ag NP-induced apoptosis was associated with the generation of ROS and mitochondria-dependent jun-*N* terminal kinase (JNK) activation (Figure 5) [172,173]. Furthermore, disruption of the mitochondrial respiratory chain by Ag NPs may cause the production of ROS and an interruption of ATP synthesis, resulting in DNA damage (Figure 5) [174]. Because Ag NPs release silver ions (Ag^+^) into aqueous solution, it is important to determine whether the observed toxicity is due to Ag NPs or Ag^+^. Investigations have shown that Ag^+^ is more toxic than Ag NPs, though Ag NPs can be considered as a Trojan horse that enters a cell and then releases Ag^+^ to damage the cell machinery [175,176]. However, according to certain other studies, both nanoparticles and dissolved Ag^+^ contributed to the observed toxic effects [177].

In conclusion, Ag NPs are toxic both *in vitro* and *in vivo*. The possible mechanisms of Ag NP-induced toxicity include Ag^+^ release, ROS generation, oxidative stress, mitochondrial damage, and the induction of apoptosis. More comprehensive studies that clearly elucidate the mechanisms of Ag NP-induced toxicity are still needed.

## 6. Iron Nanoparticles

Both zero-valent iron nanoparticles (nZVI) and iron oxide nanoparticles, such as superparamagnetic iron oxide nanoparticles (SPIONPs), have been used as catalysts for various reactions, such as Fischer-Tropsch synthesis [178], a high-temperature water-gas shift reaction [75], and the oxidation of alcohols and aldehydes [76,179]. In environmental remediation, Fe_2_O_3_ on Raschig glass rings (Fe_2_O_3_/RR) has been used as a photocatalyst for the degradation of 4-chlorophenol (4-CP) and the azo dye Orange II (Or II) [180]. Several iron oxides, including γ-Fe_2_O_3_, Fe_3_O_4_-C composite, and α-FeOOH, have been found to be useful for reducing the concentration of nitrogen (N) in fuel oil [181]. Additionally, various nZVI NPs have been applied in the degradation of organic contaminants and the remediation of inorganic contaminants [182]. While taking advantage of the potential benefits of iron nanoparticles, we also need to pay attention to the potential cellular damage associated with these nanoparticles.

Several cytotoxicity studies have shown that SPIONP exposure may result in cellular perturbations [183], gene expression alterations [184], and perturbed cell proliferation [185] in various cell lines. Similar to other NPs, surface functionalization may modulate the cytotoxicity of SPIONPs [186,187,188], with the length of the surface ligand shown to play a role. In a study of the relationship between the length of polyethylene oxide (PEO) and SPIONP-induced toxicity, NPs with the shortest ligand exhibited the highest toxicity [189]. nZVI have also been reported to be cytotoxic, and the exposure of human bronchial epithelial cells (16HBE14o) to nZVI NPs resulted in dose-dependent ROS generation and decreased cell viability [190].

The exposure to maghemite γ-Fe_2_O_3_ (0.8 mg/kg) in Wistar rats by a single intravenous injection led to toxicity in the liver, kidneys, and lungs, without affecting the brain and heart [191]. Based on animal studies and human trials, high amounts of SPIONPs may be toxic, whereas a low concentration of NPs appears to be biocompatible [192,193,194].

**Figure 6:**
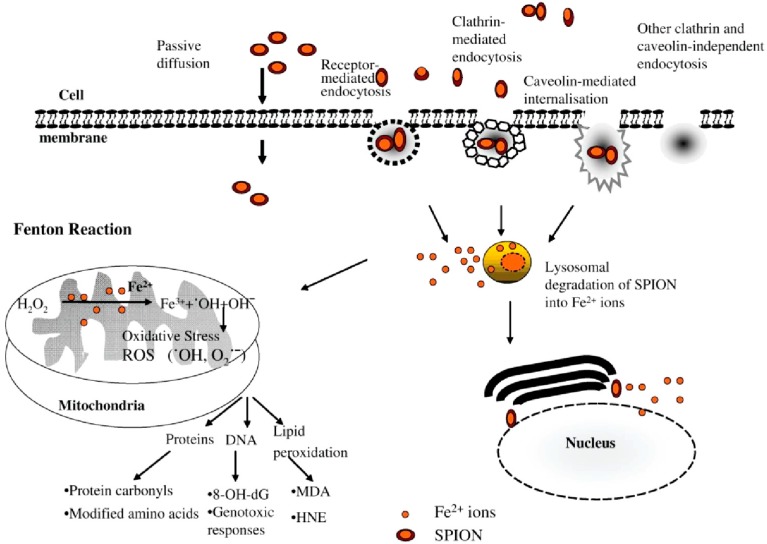
Schematic representation of the different intracellular uptake pathways of SPIONPs (superparamagnetic iron oxide nanoparticles) (8-OH-dG, 8-hydroxydeoxyguanosine; MDA, malondialdehyde; HNE, 4-hydroxy-2-nonenal). Reprinted from [195] with permission from Singh *et al.* [195], Copyright 2010.

Like other nanoparticles, SPIONPs enter cells via various pathways, such as passive diffusion, receptor-mediated endocytosis, clathrin-mediated endocytosis, caveolin-mediated internalization, and other clathrin- and caveolin-independent endocytosis mechanisms (Figure 6) [195]. Following internalization, SPIONPs are presumably degraded to iron ions within the lysosomes under the influence of hydrolytic enzymes [196,197]. These iron ions may cross the nuclear or mitochondrial membrane and generate reactive hydroxyl radicals via the Fenton reaction. These hydroxyl radicals then cause lipid peroxidation, DNA damage, and protein oxidation [30].

In short, SPIONP-induced cytotoxicity has been linked to cellular uptake and ROS generation. Although iron nanoparticles were shown to be biocompatible *in vivo* at a low concentration, these particles were found to be cytotoxic *in vitro*. An investigation using a prolonged low-dose exposure in animal models is necessary for a better understanding of the toxicity induced by this class of nanoparticles.

## 7. Conclusions

With the development of nanotechnology, a new generation of nanocatalysts has been developed and used in various areas, including environmental remediation. However, in contrast to traditional catalysts, there has been no systematic characterization of the risk hazards of nanomaterials, and there is a general lack of safety regulations for using such catalysts.

In this review, we surveyed an array of toxicity assessments of the popular nanocatalysts used in environmental remediation. In general, nearly all nanocatalysts induce toxic effects both *in vitro* and *in vivo* at a certain concentration. ROS generation and cell signaling perturbations appear to be the widely accepted causes of nanotoxicity. In addition, particle size and surface functionalization are crucial factors that determine the toxicity of nanoparticles.

Although a degree of understanding of the toxicity of popular nanocatalysts has been achieved, a comprehensive understanding, particularly of the important quantitative nanostructure-toxicity relationship, is awaiting further research. Furthermore, additional investigations in animal models and studies of composite nanocatalysts are still needed.
